# Childhood maltreatment and adult medical morbidity in mood disorders: comparison of unipolar depression with bipolar disorder

**DOI:** 10.1192/bjp.2018.178

**Published:** 2018-11

**Authors:** Georgina M. Hosang, Helen L. Fisher, Karen Hodgson, Barbara Maughan, Anne E. Farmer

**Affiliations:** 1 Senior Lecturer in Mental Illness and Chronic Diseases, Centre for Psychiatry, Wolfson Institute of Preventive Medicine, Barts and the London School of Medicine and Dentistry, Queen Mary University of London, UK; 2 Reader in Developmental Psychopathology, Social, Genetic and Developmental Psychiatry Centre, Institute of Psychiatry, Psychology & Neuroscience, King's College London, UK; 3 Postdoctoral Research Associate, Social, Genetic and Developmental Psychiatry Centre, Institute of Psychiatry, Psychology & Neuroscience, King's College London, UK; 4 Professor of Developmental Epidemiology, Social, Genetic and Developmental Psychiatry Centre, Institute of Psychiatry, Psychology & Neuroscience, King's College London, UK; 5 Emeritus Professor in Psychiatric Nosology, Social, Genetic and Developmental Psychiatry Centre, Institute of Psychiatry, Psychology & Neuroscience, King's College London, UK

**Keywords:** Bipolar affective disorders, depressive disorders, trauma, medical comorbidity

## Abstract

**Background:**

The medical burden in mood disorders is high; various factors are thought to drive this pattern. Little research has examined the role of childhood maltreatment and its effects on medical morbidity in adulthood among people with unipolar depression and bipolar disorder.

**Aims:**

This is the first study to explore the association between childhood maltreatment and medical morbidity in bipolar disorder and in unipolar depression, and examine whether the impact of abuse and neglect are distinct or combined.

**Method:**

The participants consisted of 354 psychiatrically healthy controls, 248 participants with recurrent unipolar depression and 72 with bipolar disorder. Participants completed the Childhood Trauma Questionnaire and received a validated medical history interview.

**Results:**

Any type of childhood maltreatment, child abuse and child neglect were significantly associated with the medical burden in bipolar disorder, but not unipolar depression or for controls. These associations worked in a dose–response fashion where participants with bipolar disorder with a history of two or more types of childhood maltreatment had the highest odds of having a medical illness relative to those without such history or those who reported one form. No such significant dose–response patterns were detected for participants with unipolar depression or controls.

**Conclusions:**

These findings suggest that childhood maltreatment may play a stronger role in the development of medical illnesses in individuals with bipolar disorder relative to those with unipolar depression. Individuals who had been maltreated with a mood disorder, especially bipolar disorder may benefit most from prevention and intervention efforts surrounding physical health.

**Declaration of interest:**

None.

On average people with mood disorders die up to 10 years earlier than the general population,^1^ which is largely attributed to the high medical burden in this group.^2^^,^^3^ Emerging evidence suggests that exposure to childhood adversity (for example maltreatment, abuse and neglect) is associated with medical morbidity in mood disorders.^4^^–^^6^ These studies have not distinguished between unipolar depression and bipolar disorder^5^^,^^7^ or have focused on only one disorder.^4^^,^^6^ Thus the question remains: is the influence of childhood maltreatment on adult medical morbidity more pertinent to bipolar disorder compared with unipolar depression? Moreover, previous studies have largely ignored the role of neglect, even though evidence shows it is distinct from abuse^8^ and has been linked to heart disease, osteoarthritis and other medical disorders in the general population^9^^,^^10^ and thus an important risk factor to investigate in this context. The aim of this study was to address the gaps in the literature by examining the association between childhood maltreatment and medical illnesses in adults with recurrent unipolar depression, bipolar disorder and controls. The second aim was to explore whether one type of maltreatment (i.e. child abuse or neglect) will be more pertinent to adult medical morbidity in mood disorders. Finally, a dose–response relationship between childhood maltreatment histories and the medical burden in mood disorders was investigated. Drawing on the existing literature it is postulated that childhood maltreatment will be more strongly associated with medical morbidity in bipolar disorder relative to unipolar depression with child abuse exerting the strongest influence. It is also anticipated that individuals exposed to multiple forms of maltreatment will have more medical illnesses than those who experienced one or no form of maltreatment, given a similar pattern has been observed in general population samples.^11^

## Method

### Participants

This study consisted of 674 participants from three groups: individuals with bipolar disorder, recurrent depression and controls (Table 1). The bipolar group were enrolled in the BADGE (Gene-Environmental interplay in Bipolar Affective Disorder) study^12^ where participant recruitment involved re-contacting individuals with bipolar disorder from the BaCCs (Bipolar affective disorder Case Control) study.^13^ Individuals with recurrent depression and controls were a subsample of the Depression Case-Control (DeCC) genetic association study^14^ who provided information on their experience of childhood maltreatment.

**Table 1:** Rates of medical illnesses and history of childhood maltreatment among psychiatrically healthy controls and the participants with unipolar depression and bipolar disorder

	Bipolar group, *n* (%) (*n* = 72)	Unipolar group, *n* (%) (*n* = 248)	Control group, *n* (%) (*n* = 354)	Statistic	*P*	*Post hoc* analysis, groups
Women, *n* (%)	56 (78)	183 (74)	205 (58)	χ^2^(2) = 20.33	**<0.001**	Unipolar depression, bipolar disorder > control
Age at assessment, mean (s.d.)	48.36 (9.43)	45.41 (12.76)	47.73 (9.15)	*F*(2, 639) = 4.05	**0.018**	Control, bipolar disorder > unipolar depression
Married or cohabiting, *n* (%)	36 (50)	106 (43)	–	χ^2^(1) = 0.04	0.834	–
Employed, *n* (%)	33 (46)	70 (28)	–	χ^2^(1) = 1.88	0.171	–
Number of years in education, mean (s.d.)	14.82 (3.02)	13.40 (3.48)	–	*t*(140) = 3.31	**0.0012**	Bipolar disorder > unipolar depression
Age of illness onset, years: mean (s.d.)	20.63 (14.10)	22.26 (11.33)	–	*t*(247) = 0.92	0.357	–
Duration of illness, years: mean (s.d.)	28.14 (14.18)	21.69 (13.65)	–	*t*(247) = 3.87	**<0.001**	Bipolar disorder > unipolar depression
Number of depressive episodes, mean (s.d.)	14.19 (22.49)	3.52 (3.52)	–	*t*(150) = 4.31	**<0.001**	Bipolar disorder > unipolar depression
Number of manic episodes, mean (s.d.)	12.43 (21.43)	–	–	–	–	–
At least one medical illness, *n* (%)	36 (50)	113 (46)	86 (24)	χ^2^(2) = 37.18	**<0.001**	Unipolar depression, bipolar disorder > control
Number of medical illnesses, *n* (%)						
None	36 (50)	135 (54)	268 (76)	–	–	–
1	23 (32)	78 (32)	75 (21)	–	–	–
2 or more	13 (18)	35 (14)	11 (3)	χ^2^(4) = 48.05	**<0.001**	Unipolar depression, bipolar disorder > control
Different types of medical illnesses, *n* (%)						
Heart problems (i.e. stroke, angina and heart attack)	1 (1)	11 (4)	2 (0.6)	Two-tailed Fisher's exact	**0.005**	Unipolar depression > control
Asthma	15 (21)	41 (17)	31 (9)	χ^2^(2) = 12.35	**0.002**	Unipolar depression, bipolar disorder > control
Diabetes (types 1 and 2)	5 (7)	6 (2)	7 (2)	Two-tailed Fisher's exact	0.07	–
Arthritis (i.e. osteoarthritis and rheumatoid arthritis)	11 (15)	45 (18)	23 (7)	χ^2^(2) = 20.11	**<0.001**	Unipolar depression, bipolar disorder > control
Hypertension	16 (22)	42 (17)	32 (9)	χ^2^(2) = 13.34	**0.001**	Unipolar depression, bipolar disorder > control
Epilepsy or convulsions	3 (4)	6 (2)	0 (0)	Two-tailed Fisher's exact	**0.001**	Unipolar depression, bipolar disorder > control
Osteoporosis	3 (4)	9 (4)	3 (0.9)	Two-tailed Fisher's exact	**0.022**	Unipolar depression > control
Multiple sclerosis	0 (0)	0 (0)	0 (0)	–	–	–
Childhood maltreatment, *n* (%)						
Any type of childhood maltreatment (abuse and/or neglect)^a^^,^^b^	37 (51)	136 (55)	64 (18)	χ^2^(2) = 95.74	**<0.001**	Unipolar depression, bipolar disorder > control
*Number of different childhood maltreatment histories,* n *(%)*						
None	35 (49)	112 (45)	290 (82)	–	–	–
One form of maltreatment	18 (25)	48 (19)	41 (12)	–	–	–
Two or more forms of maltreatment	19 (26)	88 (36)	23 (7)^c^	χ^2^(4) = 109.08	**<0.001**	Unipolar depression, bipolar disorder > control
Any type of child abuse^a^^,^^d^	31 (43)	111 (45)	41 (12)	χ^2^(2) = 91.46	**<0.001**	Unipolar depression, bipolar disorder > control
Physical abuse	8 (11)	33 (13)	13 (4)	χ^2^(2) = 19.42	**<0.001**	Unipolar depression, bipolar disorder > control
Emotional abuse	20 (28)	88 (36)	23 (7)	χ^2^(2) = 81.84	**<0.001**	Unipolar depression, bipolar disorder > control
Sexual abuse	20 (28)	48 (19)	20 (6)	χ^2^(2) = 39.52	**<0.001**	Unipolar depression, bipolar disorder > control
Any type of child neglect^a^^,^^e^	20 (28)	100 (40)	36 (10)	χ^2^(2) = 75.51	**<0.001**	Unipolar depression, bipolar disorder > control
Physical neglect	10 (14)	49 (20)	22 (6)	χ^2^(2) = 25.57	**<0.001**	Unipolar depression, bipolar disorder > control
Emotional neglect	19 (26)	91 (37)	28 (8)	χ^2^(2) = 75.94	**<0.001**	Unipolar depression bipolar disorder > control

*P*-values in bold are significant.

a. These figures are not the sum of the derived variables as some participants report experiencing more than one type of maltreatment.

b. Childhood maltreatment was considered present if any type of child abuse or neglect were rated as moderate or severe.

c. Percentages are greater than 100 because of rounding up of figures.

d. Child abuse was considered present if any form of child abuse was rated as moderate or severe.

e. Child neglect was considered present if physical or emotional neglect was rated as moderate or severe.

Those individuals with unipolar depression and bipolar disorder were recruited via out-patient psychiatric clinics, general practitioner surgeries, media advertisement and self-help groups in the UK (the proportion of participants recruited by each method was not recorded). Participants with a mood disorder needed to meet the DSM-IV criteria^15^ for bipolar I or II disorder (bipolar group) or have experienced two or more depressive episodes of moderate severity (unipolar group). The exclusion criteria included intravenous drug use with a lifetime diagnosis of drug dependency, mood episodes that only occurred as a result of substance misuse or physical illness, or personal or family history of schizophrenia or mania (unipolar group only). At the time of their assessments (BaCCs or BADGE) the individuals with bipolar disorder were not experiencing a mood episode.

Controls were recruited through general practices and excluded if they had a personal or a first-degree relative with a history of mental illness. All participants were White to minimise population stratification because they were originally recruited for genetic studies. Participants were 18 years or over and provided written informed consent. All studies received ethical approval from local university and National Health Service ethics committees (BADGE: King's College Hospital Ethics Committee (ref: 06/Q0703/250); the Joint South London and Maudsley, and Institute of Psychiatry Research Ethics Committee approved DeCC (ref: 195/00) and BaCCs (ref: 187/02). The procedures used in these studies were in line with the Declaration of Helsinki in 1975 (revised in 2008), as well as national and institutional ethical committee standards of human experimentation.

### Measures

#### Psychiatric diagnoses

The Schedules for Clinical Assessment in Neuropsychiatry (SCAN)^16^ interview was administered to obtain a lifetime DSM-IV diagnosis of either bipolar disorder or depression (dependent on group).^15^ The presence and severity of the SCAN items were rated for the two most severe depressive episodes for the participants with recurrent depression. For individuals with bipolar disorder, the worst depressive and manic episodes were the focus of the interview. The SCAN interview also enquired about the number of years in education, marital and employment status as well as age of mood disorder onset and number of mood disorder episodes experienced.

#### History of childhood maltreatment

The Childhood Trauma Questionnaire (CTQ)^17^ assessed the experience of five forms of maltreatment, covering child abuse (sexual, emotional and physical abuse) and neglect (emotional and physical neglect). The CTQ consists of 28 items rated on a five-point Likert scale ranging from ‘never true’ to ‘very often true’, five items were used to assess each form of maltreatment. Cut-offs for moderate to severe levels of each form of maltreatment were used in this investigation based on the CTQ manual,^17^ these were then grouped into two categories: neglect and abuse. The CTQ has very good psychometric properties.^17^

#### Medical history

The lifetime diagnosis of various medical disorders was measured using a self-report questionnaire that was administered to all participants by trained research assistants.^18^^,^^19^ Participants reported whether they had received a formal diagnosis by a health professional (for example general practitioner) of these illnesses: heart problems (stroke, angina and heart attack), asthma, diabetes (type 1 and type 2), arthritis (osteoarthritis and rheumatoid arthritis), hypertension, epilepsy or convulsions, osteoporosis or multiple sclerosis. High levels of similarity were found between the self-report of medical disorders using this instrument and health practitioner ratings in a subset of this sample.^18^

### Analyses

Differences between groups were tested using χ^2^ tests, one-way ANOVAs or independent samples *t*-tests dependent on the data being analysed. In instances where the χ^2^ test could not be used (expected values were less than five) Fisher's exact tests were employed. Two approaches were used to examine differences between mood disorder status (i.e. bipolar disorder, unipolar depression and control) in the relationship between childhood maltreatment and medical disorders. When the focus was on at least one medical illness, logistic regression models were conducted and ordinal logistic regression models were used for the number of medical disorders (none, one and two or more illnesses). The variables entered into the models consisted of childhood maltreatment or number of forms of maltreatment (none, one, and two or more; when focused on the impact of polyvictimisation), mood disorder status and the interaction between the two variables; gender and age were included as covariates. To examine the effect of the different types of childhood maltreatment histories (any form of maltreatment, child neglect and child abuse) three parallel models were undertaken. The analyses were re-run adjusting for age at mood disorder onset and duration of illness for mood disorder (presented in the tables as model 2). The significance level of *P* ≤ 0.05 was adopted in this investigation. The analyses were undertaken using Stata version 13.1.

## Results

Participants in the unipolar group were significantly younger than those in the bipolar group (*P* = 0.035) and the control group (*P* = 0.012), according to a Tukey *post hoc* test. A significantly higher proportion of those in the bipolar and unipolar groups were women relative to the control group. The mood disorder groups were mainly similar based on socioeconomic status. However, the bipolar group spent significantly longer in education, had significantly longer illness duration and more depressive episodes than their counterparts with unipolar depression (Table 1).

The lifetime prevalence of the different medical illnesses and childhood maltreatment histories are presented in Table 1 and are organised by group. The most frequently reported medical disorders in the overall sample were hypertension, arthritis and asthma. The low frequency of each medical disorder precluded the exploration of associations between specific illnesses and childhood maltreatment. The subsequent analyses focused on the diagnosis of ‘at least one’ or the ‘number of’ (none, one and two or more) medical disorders. The prevalence of the different types of childhood maltreatment were significantly higher among the bipolar and unipolar groups compared with the control group. The most frequently reported forms of maltreatment were emotional abuse and neglect. Any type of abuse was moderately correlated with any type of neglect in the whole sample (Pearson's *r* (674) = 0.47, *P* < 0.001).

### What factors are associated with having a medical illness?

A similar proportion of men and women reported being diagnosed with at least one (χ^2^(1) = 0.03, *P* = 0.869) or a greater number of medical disorders (χ^2^(2) = 0.03, *P* = 0.986). Participants that reported being diagnosed with at least one medical illness were significantly older compared with those without a diagnosis (*t*(640) = 6.20, *P* < 0.001). A comparable result was found when the number of medical disorders were examined (*F*(2, 639) = 24.93, *P* < 0.001). Specifically, individuals that reported being diagnosed with two or more medical illnesses were significantly older (mean 54.23, s.d. = 9.47) than those that reported the diagnosis of one (mean 49.03, s.d. = 11.09) or no medical illnesses (mean 45.00, s.d. = 10.20), based on a Tukey *post hoc* test (*P* < 0.001).

The effects of socioeconomic status, clinical variables, childhood maltreatment and mood disorder diagnosis on having a medical illness are presented in Table 2. None of the socioeconomic status indices were associated with being diagnosed with at least one or a greater number of medical disorders. In terms of clinical characteristics, illness duration of mood disorder and younger age of mood disorder onset were significantly associated with the medical burden in mood disorders.

**Table 2:** Main and interaction effects of mood disorder status and childhood maltreatment on diagnoses of medical illnesses

	Model 1, adjusted for gender and age			Model 2, adjusted for age, gender, illness duration and age of mood disorder onset		
	OR	95% CI	*P*	OR	95% CI	*P*
*At least one medical illness*						
Main effects						
Bipolar disorder diagnosis	3.12	1.81–5.42	**<0.001**	–	–	–
Unipolar depression diagnosis	3.05	2.08–4.46	**<0.001**	–	–	–
Childhood maltreatment^a^	2.15	1.52–3.05	**<0.001**	–	–	–
** ** Child abuse^b^	2.35	1.62–3.41	**<0.001**	–	–	–
** ** Child neglect^c^	2.48	1.68–3.65	**<0.001**	–	–	–
Married or cohabiting	0.93	0.55–1.55	0.770	–	–	–
Years in education	0.93	0.84–1.03	0.150	–	–	–
Employed	0.70	0.41–1.20	0.198	–	–	–
Clinical characteristics of mood disorder:						
Age of mood disorder onset	0.96	0.94–0.99	**0.002**	–	–	–
Duration of illness for mood disorder	1.04	1.02–1.07	**0.001**	–	–	–
Number of depressive episodes	1.01	0.99–1.03	0.281	–	–	–
Interaction effects						
Bipolar disorder × childhood maltreatment	7.64	2.21–26.41	**0.001**	6.13	1.78–21.07	**0.004**
Bipolar disorder × abuse	9.13	2.39–34.97	**0.001**	3.01	0.94–9.63	0.063
Bipolar disorder × neglect	4.14	0.99–17.37	0.052	3.58	1.10–11.64	**0.034**
Unipolar depression × childhood maltreatment	1.38	0.59–3.21	0.455	0.98	0.51–1.86	0.939
Unipolar depression × abuse	1.96	0.74–5.17	0.173	1.05	0.56–2.02	0.862
Unipolar depression × neglect	1.24	0.48–3.23	0.660	1.26	0.66–2.43	0.482
*Number of medical illnesses*						
Main effects						
Bipolar disorder diagnosis	3.57	2.10–6.09	**<0.001**	–	–	–
Unipolar depression diagnosis	3.31	2.28–4.79	**<0.001**	–	–	–
Childhood maltreatment^a^	2.19	1.57–3.07	**<0.001**	–	–	–
** ** Child abuse^b^	2.54	1.78–3.63	**<0.001**	–	–	–
** ** Child neglect^c^	2.58	1.78–3.73	**<0.001**	–	–	–
Married or cohabiting	0.97	0.60–1.58	0.904	–	–	–
Years in education	0.93	0.84–1.02	0.118	–	–	–
Employed	0.65	0.39–1.09	0.099	–	–	–
Clinical characteristics of mood disorder:						
Age of mood disorder onset	0.97	0.94–0.99	**0.004**	–	–	–
Duration of illness for mood disorder	1.04	1.01–1.06	**0.003**	–	–	–
Number of depressive episodes	1.02	1.00–1.04	0.128	–	–	–
Interaction effects						
Bipolar disorder × childhood maltreatment	7.18	2.23–23.08	**0.001**	4.95	1.66–14.73	**0.004**
Bipolar disorder × abuse	8.70	2.49–30.33	**0.001**	3.14	1.06–9.32	**0.039**
Bipolar disorder × neglect	3.58	1.01–12.73	**0.049**	3.56	1.26–10.11	**0.017**
Unipolar depression × childhood maltreatment	1.36	0.60–3.10	0.460	1.08	0.59–1.98	0.800
Unipolar depression × abuse	1.91	0.74–4.92	0.183	1.13	0.62–2.08	0.690
Unipolar depression × neglect	1.40	0.55–3.54	0.476	1.54	0.84–2.85	0.166

OR, odds ratio derived from binary logistic regression for ‘at least one medical illness’ and from ordinal logistic regression for ‘number of medical illnesses’.

a. Childhood maltreatment was considered present if any type of child abuse or neglect were rated as moderate or severe.

b. Child abuse was considered present if any form of child abuse was rated as moderate or severe.

c. Child neglect was considered present if physical or emotional neglect was rated as moderate or severe.

Having a history of childhood maltreatment, child abuse and neglect more than doubled the odds of having at least one or a greater number of medical disorders. Having a medical illness was also significantly associated with a diagnosis of unipolar depression or bipolar disorder.

### Does the association between childhood maltreatment and having a medical illness differ between the mood disorder groups?

**Fig. 1:**
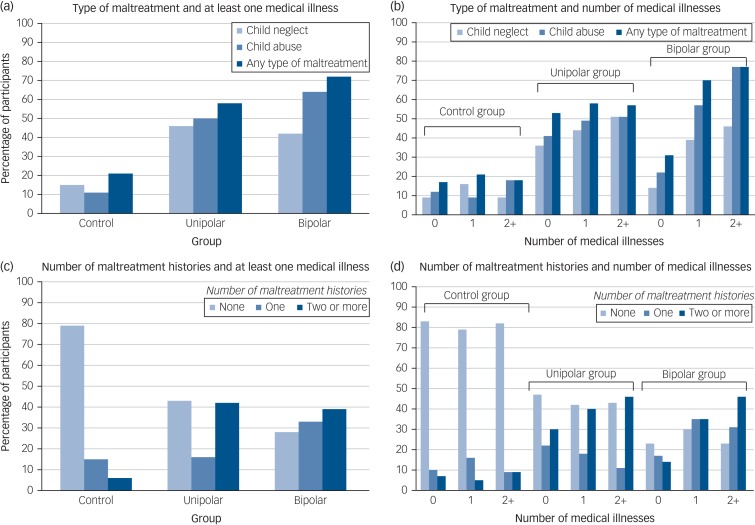
Percentage of participants from each group with medical illnesses by the type of maltreatment ((a) and (b)) and number ((c) and (d)) of childhood maltreatments experienced. The *y*-axis presents the percentage of participants with medical illnesses by the number (none, one, or two or more) and type (child neglect, child abuse and any type of childhood maltreatment) of childhood maltreatment recorded for the control, unipolar depression and bipolar disorder groups.

Bipolar disorder diagnosis was found to significantly interact with any form of childhood maltreatment, child abuse and child neglect on the diagnosis of at least one and a greater number of medical illnesses relative to controls (Table 2). No significant interactions were detected for unipolar depression compared with controls. The proportion of each group with medical illnesses that reported experiencing different forms of childhood maltreatment is visually presented in Fig. 1.

To test whether the clinical expression of mood disorders was driving the results the analyses were re-run adjusting for duration of illness for mood disorder and age of mood disorder onset (model 2). Although the results were attenuated for bipolar disorder they remained significant with odds ratios (ORs) of at least 3.

### Does the relationship between childhood maltreatment and medical morbidity work in a dose–response fashion?

The number of different types of childhood maltreatment (emotional abuse, physical abuse, sexual abuse, emotional neglect, and/or physical neglect) each participant reported was coded as none (0), one (1) or two or more (2). For the bipolar group the odds for being diagnosed with at least one or a greater number of medical illnesses was highest among those who had been exposed to two or more types of maltreatment (at least one medical illness: adjusted OR = 5.45, 95% CI 1.30–22.85, *P* = 0.020; number of medical disorders: adjusted OR = 5.13, 95% CI 1.42–18.50, *P* = 0.012), followed by the those who were subject to one form (at least one medical illness: adjusted OR = 4.71, 95% CI 1.30–17.09, *P* = 0.019; number of medical illnesses: adjusted OR = 4.26, 95% CI 1.31–13.84, *P* = 0.016) compared with those without a history of childhood maltreatment. When the results were adjusted for duration of illness for mood disorder and age of mood disorder onset (model 2) a graded relationship between number of maltreatment histories and medical morbidity was no longer observed, whereby the odds of having a medical illness was similar for those who reported one or two or more forms of maltreatment (Table 3).

**Table 3:** Dose–response relationship between childhood maltreatment and medical illnesses by mood disorder status

	Bipolar disorder group (*n* = 72)			Unipolar depression group (*n* = 248)			Control group (*n* = 354)		
	Adjusted OR^a^	95% CI	*P*	Adjusted OR^a^	95% CI	*P*	Adjusted OR^a^	95% CI	*P*
*At least one medical illness*									
Number of childhood maltreatment histories									
None (reference)	–	–	–	–	–	–	–	–	–
One	4.71	1.30–17.09	**0.019**	0.68	0.34–1.35	0.270	0.81	0.36–1.86	0.622
Two or more	5.45	1.30–22.85	**0.020**	1.71	0.94–3.12	0.078	0.50	0.15–1.69	0.266
Adjusted for age of mood disorder onset and illness duration									
None (reference)	–	–	–	–	–	–	–	–	–
One	5.24	1.19–22.89	**0.028**	0.75	0.34–1.63	0.463	–	–	–
Two or more	5.07	1.11–23.18	**0.036**	1.12	0.54–2.32	0.759	–	–	–
*Number of medical illnesses*									
Number of childhood maltreatment histories									
None (reference)	–	–	–	–	–	–	–	–	–
One	4.26	1.31–13.84	**0.016**	0.80	0.40–1.61	0.528	0.77	0.34–1.74	0.532
Two or more	5.13	1.42–18.50	**0.012**	1.71	0.98–2.98	0.060	0.52	0.16–1.70	0.275
Adjusted for age of mood disorder onset and illness duration									
None (reference)	–	–	–	–	–	–	–	–	–
One	4.52	1.24–16.47	**0.022**	0.70	0.33–1.47	0.340	–	–	–
Two or more	4.10	1.05–15.93	**0.042**	1.32	0.65–2.60	0.431	–	–	–

OR, odds ratio derived from binary logistic regression for ‘at least one medical illness’ and from ordinal logistic regression for ‘number of medical illnesses’.

a. Adjusted for the effects of gender and age.

For the participants with unipolar depression the odds of being diagnosed with at least one (OR = 1.71, 95% CI 0.94–3.12, *P* = 0.078) or a greater number (OR = 1.71, 95% CI 0.98–2.98, *P* = 0.060) of medical disorders was highest among those who reported multiple forms of maltreatment, but these associations were non-significant. However, the odds of having a medical illness was lower for those in the unipolar group that reported one type of maltreatment (at least one medical illness: adjusted OR = 0.68, 95% CI 0.34–1.35, *P* = 0.270; number of medical illnesses: adjusted OR = 0.80, 95% CI 0.40–1.61, *P* = 0.528). For the controls no such dose–response relationship was observed (Table 3). The percentage of each group with medical illnesses is presented by the number of different types of maltreatment reported in Fig. 1.

## Discussion

This is the first study to contrast the childhood maltreatment–medical morbidity relationship in bipolar disorder and unipolar depression. Our results show that any form of childhood maltreatment, child abuse and child neglect was associated with medical burden in mood disorders, but was only significant for bipolar disorder not unipolar depression. The available evidence on this topic is conflicting with one study finding a significant relationship between childhood adversity and medical comorbidities in mood disorders,^5^ whereas another failed to detect any significant associations.^7^ Several factors may explain these disparate findings, including differences in the types of childhood adversities (maltreatment, parental psychiatric illness and witnessing domestic violence) and physical illnesses (metabolic syndrome versus two or more physical illnesses) examined.^5^^,^^7^ The specific effects of mood disorder diagnosis (unipolar depression and bipolar disorder) were not investigated in these studies and may have contributed to the inconsistent findings.^5^^,^^7^ It should be noted that in the current investigation there were no significant differences in the rates of medical illnesses and the prevalence of childhood maltreatment between participants with unipolar depression and bipolar disorder, and therefore this cannot explain the observed results.

The findings in this study that maltreatment was not significantly associated with medical morbidity in unipolar depression adds to the divergent literature in this area,^5^^,^^7^ but are consistent with findings from the World Health Organization World Mental Health Surveys initiative, which consisted of data from 10 countries from the Americas, Europe and Asia.^10^ The results from this initiative showed that childhood adversities and early-onset mental illness (major depression and anxiety disorders) were independently associated with chronic physical conditions (for example heart disease and asthma).^10^^,^^20^ But found no evidence that the relationship between depression/anxiety and physical ill health was a function of a shared history of childhood adversities.^10^^,^^20^ Together these results indicate that alternative pathways (other than via childhood adversity) through which depression and physical ill health are related need to be investigated further.

Several pathways may explain why the childhood maltreatment–medical morbidity association is stronger for bipolar disorder relative to unipolar depression. First, a history of childhood maltreatment is associated with a more severe clinical presentation for both mood disorders,^21^^,^^22^ but for bipolar disorder this also includes greater risk of experiencing psychotic symptoms.^23^ Antipsychotic medications are commonly used to treat psychotic symptoms among people with bipolar disorder, which may be prescribed in conjunction with mood stabilisers.^24^ The adverse effects of antipsychotic medications (compared with mood stabilisers and antidepressants) and polypharmacy (relative to monopharmacy) have been shown to be more strongly linked to physical ill health.^25^ Thus, it is possible that the link between childhood maltreatment and medical morbidity is more relevant to bipolar disorder via psychosis and side-effects of relevant pharmacological treatment.

The relationship between childhood maltreatment and medical illnesses may be influenced by other clinical variables that are more pertinent to bipolar disorder than unipolar depression. For instance, when we included clinical variables (such as illness duration) in the analyses we found that the associations between maltreatment and having a medical illness were attenuated. Duration of untreated illness is particularly long for bipolar disorder with a range of 5–10 years.^26^ Longer duration of untreated illness has been associated with childhood maltreatment^27^ and having a medical illness^28^ in bipolar samples, therefore it would be an important variable to consider in future studies examining the maltreatment–medical morbidity relationship in bipolar disorder.

Second, people with bipolar disorder have been found to exhibit significantly higher inflammation levels compared with those with unipolar depression.^29^ The magnitude of these differences may be greater when combined with exposure to childhood maltreatment, which is also linked to elevated inflammation.^30^ It is plausible that this combined effect could give rise to especially pronounced inflammation levels, further increasing the chances of developing a medical illness among people with bipolar disorder rather than those with unipolar depression. This should be directly examined in future studies.

The findings from the current investigation concerned with the specific effects of child abuse and neglect on the medical burden in unipolar depression compared with bipolar disorder present a novel contribution to the literature. Previous studies have either not investigated the specific impact of child neglect in this context and/or have not distinguished between bipolar disorder and unipolar depression.^5^^,^^6^ The results of the present investigation indicate that child abuse exerts a stronger effect on adult medical morbidity in mood disorders relative to child neglect. These findings are similar to the results of general population-based studies, which show that child abuse is associated with a constellation of adult medical disorders whereas child neglect is linked to a restricted number of illnesses.^9^^,^^31^ Obesity is a key risk factor for various medical illnesses, ranging from type 2 diabetes to cardiovascular disease.^32^ The results from a recent meta-analysis show that all types of child abuse but not emotional neglect are significantly associated with obesity in adulthood.^32^ The causal pathways behind the child abuse–medical illness link in adults with mood disorders need to be illuminated with further research.

The findings of the current study also revealed that an accumulation of childhood maltreatment had a stronger impact on the medical burden in mood disorders in adulthood. These findings replicate those reported in the large community based sample from the Adverse Childhood Experiences study, which showed graded relationships between the number of adversities experienced and adult medical illnesses (such as cancer and liver disease) and poor mental health;^33^^,^^34^ patterns that have been confirmed in a recent meta-analysis.^11^ In the current study we found that participants with a mood disorder and a history of multiple forms of maltreatment were found to have the highest odds of being diagnosed with a medical illness relative to those with one or no history, although these associations were only significant for bipolar disorder. The experience of multiple forms of maltreatment may indicate a greater burden on the individual because of repeated activation of the stress response increasing the susceptibility to poor health outcomes.^35^ A more detailed enquiry of the experience of childhood maltreatment would specifically address this assertion.

Among the participants with unipolar depression we also found that experiencing one form of maltreatment was associated with a lower odds of having a medical illness, although this pattern was non-significant. These findings are in contrast to that of previous studies,^5^^,^^10^ which have examined a spectrum of adversities occurring in childhood (for example parental psychopathology) whereas the focus here was on maltreatment. Further research in this area is warranted to further illuminate the relationship between polyvictimisation and medical burden in unipolar depression.

### Implications

The findings of this study have several possible clinical implications. First, the results underscore the high medical morbidity in mood disorders, especially bipolar disorder, highlighting the need for regular physical health assessments in affected individuals. These assessments will likely have a positive impact on people with mood disorders since they will permit earlier detection of medical illness. This would mean timely implementation of prevention and intervention strategies improving the prognosis and reducing the morbidity and mortality attributed to physical illnesses. Second, with replication the findings of the current investigation will help to identify a subgroup (those with a history of maltreatment) of people with mood disorders, who may be especially vulnerable to a worse clinical course and poor physical health. Since this group is at increased risk for these negative outcomes they would benefit most from prevention and intervention work and should be deliberately targeted.

### Methodological considerations

This study benefits from using standardised measures of childhood maltreatment and physical health, administered to well-characterised controls, individuals with recurrent unipolar depression and bipolar disorder. But it is also subject to several limitations that should be considered when interpreting the findings. First, this study included a modest number of participants with bipolar disorder, which may have had an impact on the results, although the findings are comparable with those of a larger investigation consisting of over 900 participants with bipolar disorder.^6^

Another methodological issue is the reliance on self-report measures of childhood maltreatment and medical history, which are vulnerable to several biases that could lead to inaccuracies in the information reported.^36^ Retrospective self-report childhood maltreatment questionnaires are frequently used in general population and clinical studies for convenience,^22^^,^^31^ although they may miss some individuals who have been maltreated who would have been identified using prospective measures.^37^ Nonetheless, substantial concordance between the information provided from self-report childhood maltreatment questionnaires and therapist ratings^17^ have been found, demonstrating convergent validity. High agreement between the self-report medical assessment used in the current study and physician reports of the diagnosis of physical illnesses have been reported.^18^

Differences between the unipolar and bipolar groups in this study may have contributed to the non-significant association between maltreatment and medical burden in unipolar depression. For instance, individuals with bipolar disorder reported significantly longer illness duration compared with the participants with unipolar depression. Longer illness duration for mood disorders has been associated with worse physical health outcomes^19^ and may have confounded the association between childhood maltreatment and medical illnesses in mood disorders reported here. Although the results were adjusted for illness duration it would be beneficial for future studies on this topic to clinically match participants with bipolar disorder and unipolar depression to reduce the confounding potential of clinical factors.

Demographic characteristics of the study sample may have influenced the results. For instance, all of the participants in the current investigation were White, therefore it is unclear whether the results reported here are generalisable to other ethnic groups. Given the well documented ethnic disparities in the rates of various medical illnesses (such as type 2 diabetes)^38^ it is important to consider this variable in the context of childhood maltreatment and its association with physical health in adulthood.

A history of childhood maltreatment has been significantly linked to alcohol and substance use disorders,^9^ which are key risk factors for poor health outcomes.^39^ In the present study only participants without a history of substance and alcohol dependence were included, thus we are unable to assess the influence of these histories on the childhood maltreatment–medical morbidity in mood disorders and this should be considered in subsequent research. It is important to note that the study exclusion criteria also listed mood episodes only occurring as a result of physical illnesses, this may have had an impact on the study's ability to truly test the association between childhood maltreatment and the medical burden in mood disorders and should be addressed in future research endeavours.

Future studies designed to address the limitations outlined here would be of great value, specifically they should use large ethnically diverse cohorts of individuals with bipolar disorder and unipolar depression that prospectively report on their experience of childhood maltreatment combined with physician ratings of participants' medical history and assessment of alcohol and substance use disorders. It would be also be useful for future studies to investigate a broader construct of childhood adversity that included additional indicators, such as household dysfunction (for example exposure to domestic violence) and other types of victimisation (such as bullying), especially given the interrelated nature of such experiences.^8^

In sum, this is the first investigation to examine the relationship between childhood maltreatment and adult medical illnesses in bipolar disorder compared with unipolar depression. The findings showed that any form of childhood maltreatment, child abuse and child neglect was significantly related to the diagnosis of at least one and a greater number of medical illnesses in bipolar disorder but not unipolar depression. These associations worked in a graduated fashion where individuals with bipolar disorder with a history of two or more types of childhood maltreatment had the highest odds of having a medical illness, compared with those who reported one or no form of maltreatment. No such significant dose–response patterns were detected for those in the unipolar or control groups. These results indicate that individuals with bipolar disorder who have experienced childhood maltreatment are more likely to have a medical illness and would benefit most from prevention and intervention efforts surrounding physical health.
